# Lineage origin and transcriptional control of autoantigen-specific T-regulatory type 1 cells

**DOI:** 10.3389/fimmu.2023.1267697

**Published:** 2023-09-25

**Authors:** Edgar Angelats, Pere Santamaria

**Affiliations:** ^1^ Pathogenesis and Treatment of Autoimmunity Group, Institut D’Investigacions Biomèdiques August Pi i Sunyer, Barcelona, Spain; ^2^ Department of Microbiology, Immunology and Infectious Diseases, Snyder Institute for Chronic Diseases, Cumming School of Medicine, University of Calgary, Calgary, AB, Canada

**Keywords:** autoimmunity, T-regulatory (Treg) cells, T-regulatory type 1 (TR1) cells, peptide-MHC, nanomedicine, T-follicular helper cells (Tfh)

## Abstract

T Regulatory type-1 (TR1) cells represent an immunosuppressive T cell subset, discovered over 25 years ago, that produces high levels of interleukin-10 (IL-10) but, unlike its FoxP3+ T regulatory (Treg) cell counterpart, does not express FoxP3 or CD25. Experimental evidence generated over the last few years has exposed a promising role for TR1 cells as targets of therapeutic intervention in immune-mediated diseases. The discovery of cell surface markers capable of distinguishing these cells from related T cell types and the application of next generation sequencing techniques to defining their transcriptional make-up have enabled a more accurate description of this T cell population. However, the developmental biology of TR1 cells has long remained elusive, in particular the identity of the cell type(s) giving rise to *bona fide* TR1 cells *in vivo*. Here, we review the fundamental phenotypic, transcriptional and functional properties of this T cell subset, and summarize recent lines of evidence shedding light into its ontogeny.

## Introduction

The TR1 cell subset has been implicated in the maintenance of peripheral tolerance against immune-mediated pathologies. TR1-like cells were first documented in severe combined immunodeficiency (SCID) patients that did not develop graft-versus-host disease (GvHD) after receiving HLA-mismatched fetal liver hematopoietic stem cell transplants (1). Subsequent work by Groux et al. using antigen-activated CD4^+^ T cells cultured in the presence of IL-10 led to the identification of a distinct T cell subset, thereafter named TR1, that could prevent the development of experimental colitis in an IL-10- and transforming growth factor beta (TGFβ)-dependent manner (2).

Unfortunately, the paucity of information on TR1 cell-specific surface markers or transcription factors have hampered the execution of detailed studies on the role and function of this T cell subset in the maintenance or breakdown of self tolerance. The use of relatively non-specific markers of TR1 cell identity, leading to the implication of this subset in various immunological processes (i.e., sometimes relying exclusively on IL-10 expression), has muddied progress in this area. Fortunately, the last decade has witnessed the discovery of phenotypic and molecular features of ‘TR1-ness’ that have allowed a better definition of TR1-like cells in various experimental settings. These developments, coupled to recent methodological developments in *in vitro* TR1 cell generation (3–7), and the discovery of pharmacological approaches capable of eliciting the formation and expansion of antigen-specific TR1 cells *in vivo* (8), have exposed TR1 cells as attractive targets for therapeutic intervention in immune-mediated diseases.

Despite this progress, significant knowledge gaps remain, including a detailed understanding of the developmental biology processes responsible for the genesis of this T cell subset *in vivo*. The use of novel technologies, including mass cytometry and next-generation-sequencing to address these various gaps are beginning to shed light into these areas of scientific inquiry. In this review, we summarize current knowledge on the phenotypic and molecular hallmarks of TR1 cells and key developmental processes underlying TR1 cell genesis, including recent evidence pointing towards T follicular helper (Tfh) cells as TR1 cell precursors (9, 10).

## Phenotype

TR1 cells were initially described as CD4^+^ T cells producing high levels of IL-10 and IL-5, intermediate levels of TGFβ and INFγ and low levels of IL-4 and IL-2, and were capable of suppressing specific immune responses *in vitro* and *in vivo*, in an IL-10-dependent manner (2). With rapid IL-10 production kinetics, detectable even 4 hours post-activation, and a peak of production at 24h (11), IL-10 became the hallmark cytokine for the TR1 population and, together with the absence of FoxP3 expression, used to identify TR1 cells in early studies. We now know that these criteria are insufficient, given that other CD4^+^ T cell types such as Th1 (12, 13) or Th2 (14) can produce IL-10 and acquire immunoregulatory properties; such cells do not belong to the TR1 subset. For example, Lönnberg et al. claimed a Th1 origin for TR1 cells in a chronic *Plasmodium* infection model, solely on the basis of presence of IL-10-expressing cells within the infection-induced Th1 pool, and on the assumption that TR1 cells are simply IL-10/IFNγ-co-expressing cells (15). In fact, further transcriptomic analyses of the IL-10^+^ and IL-10^–^ Th1 cells of these mice revealed the presence of only two differentially expressed genes between these subsets (*Trib2* and *BC017643*). In another study, also in a chronic *Plasmodium* infection model, Soon et al. reported a similar outcome; 34% of Th1 lineage cells co-expressed *Ifng* and *Il10* (16). It is thus likely that, based on the evidence provided, the IL-10^+^ cells that arose in these mice were IL-10-expressing Th1 cells, rather than true TR1 cells. This indicates that the assignment of TR1ness cannot merely rely on IL-10 expression.

In light of these challenges, hampering progress in defining the significance of the TR1 cell subset in both physiology and pathology, extensive efforts were made to better describe the molecular hallmarks of the TR1 subset [Reviewed in (17)]. Notwithstanding the fact that markers strictly unique to TR1 cells remain elusive, recent advances have made it possible to more accurately identify such cells in biological samples.

Gagliani and colleagues identified co-expression of CD49b and Lymphocyte-activation gene 3 (LAG-3) as surface markers for both human and murine IL-10-producing TR1-like cell populations (18). Subsequent studies indicated that a significant fraction of these IL-10-producing CD49b^+^LAG-3^+^ T cells are co-inhibitory receptor-rich, expressing Programmed cell death-1 (PD-1), T cell immunoreceptor with Ig and ITIM domains (TIGIT), T-cell immunoglobulin and mucin-domain containing protein-3 (TIM-3), and Cytotoxic T-Lymphocyte antigen 4 (CTLA-4), and co-express the co-stimulatory molecule ICOS (Inducible Costimulator) and the chemokine receptor CCR5, among other molecules (7). In agreement with these observations, intestinal TR1-like cells expressing PD-1 and CCR5 were found to co-express CD49b and LAG-3 by others (19), thus supporting the use of such markers for TR1-like cell identification. Thus, as proposed elsewhere (20), TR1 cell annotation should meet the following four criteria: 1) high IL-10 production competency (co-expression of other cytokines in variable amounts depending of environmental cues is possible); 2) immunoregulatory activity; 3) absence of constitutive FoxP3 expression (expression of FoxP3 upon activation, particularly in human TR1 cells, is not an exclusion criterium); and 4) co-expression of CD49b and LAG-3 in the presence of other co-inhibitory receptors such as PD-1, TIGIT, TIM-3 or CTLA-4 among others.

Indeed, the profoundly immunoregulatory antigen-specific TR1-like cells that arise *in vivo* in response to systemic delivery of nanoparticles (NPs) coated with mono-specific disease-relevant peptide-Major Histocompatibility Complex class II (pMHCII) molecules (8, 21–23) lack FoxP3 expression and upregulate many of the markers mentioned above, including CD49b and the co-inhibitory receptors LAG-3, PD-1, TIGIT and CTLA-4, the co-stimulator ICOS, the cytokines IL-10, IL-21 and IFNγ and the chemokine receptors CCR5 and CXCR3 (9). We have shown that administration of these compounds can lead to the resolution of inflammation in various organ-specific autoimmune disease models in a disease-specific manner without impairing normal immune responses (8, 21, 22). Adoptive transfer experiments demonstrated that the cognate (pMHCII tetramer^+^) T cells arising in these mice in response to therapy were largely, albeit not exclusively, responsible for the therapeutic properties of these compounds (TR1 cell-induced B-regulatory cells also contributed to disease suppression) (8, 22).

## Mechanisms of action

**Figure 1:**
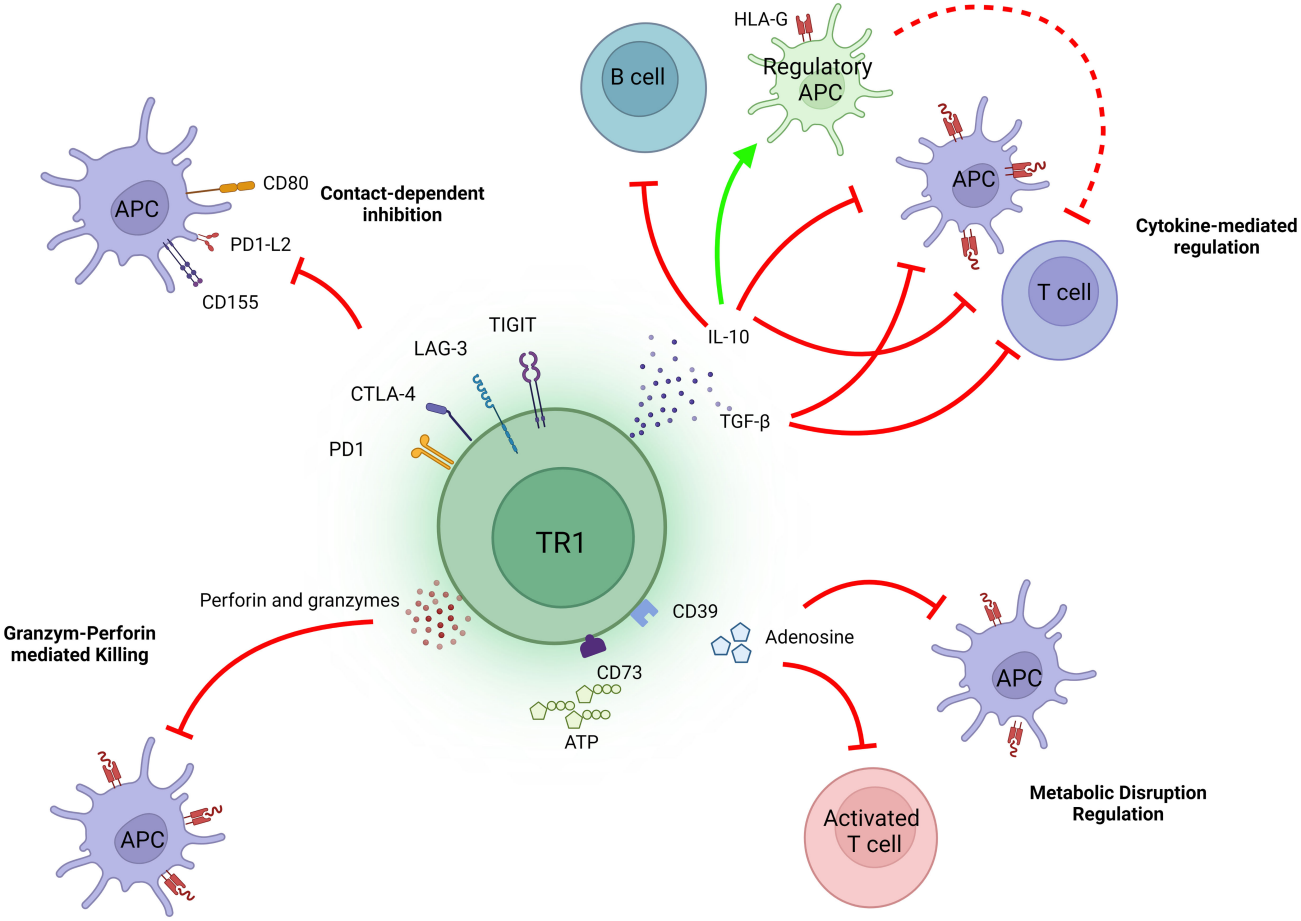
Mechanisms of action of TR1 cells. The immunosuppressive activity of TR1 cells is largely, albeit not exclusively, mediated through the release of IL-10 and TGFβ. Both cytokines can directly inhibit effector T cells and APCs, the latter also having a large indirect impact on effector T cell function. Additionally, IL-10 can imprint regulatory properties in APCs, such as by promoting the upregulation of tolerogenic molecules like HLA-G. The co-inibitory molecules expressed on TR1 cells, including CTLA-4, PD-1, TIGIT and LAG-3, can also result in contact-dependent inhibition of APCs and consequently, inhibit APC-induced T cell activation. Upon recognition of cognate pMHCII complexes on APCs, TR1 cells can also kill these cells via perforin and granzymes. In addition, these TR1 cells can inhibit T cell function by producing adenosine.

TR1 cells need to be activated to immunoregulate. Upon recognition of their cognate pMHCII complexes on co-stimulation-competent APCs, TR1 cells become productively activated. By actively inhibiting the antigen-presentation and pro-inflammatory properties of these APCs (in addition to direct effects on other target cell types, see below), TR1 cells can suppress both cognate and non-cognate effector T cell activation (a process referred to as ‘bystander immunoregulation’). This biological activity involves the deployment of several mechanisms (**Figure 1**).

### Production of immunoregulatory cytokines

As noted above, productive activation of TR1 cells leads to rapid and robust production of IL-10, which can suppress the function of different immune cell subsets, such as T cells, APCs and B cells. IL-10 can inhibit the proliferation of, and downregulate the production of effector cytokines by, effector T cells (24), and can induce an anergic state in T cells in a STAT3-dependent manner (25). Likewise, IL-10 can inhibit the production of pro-inflammatory mediators by professional APCs, and downregulate the expression of MHC class II molecules and co-stimulatory molecules on their surface (26). It can also promote the upregulation of the immunoglobulin-like transcripts 3 and 4 (ITL3 and 4) and HLA-G, which have been implicated in the generation of tolerogenic dendritic cells (DCs) (27). On B cells, IL-10 promotes proliferation, expression of MHC class II molecules and isotype switching to IgG4 (28).

TGFβ has also been implicated in TR1-mediated immunoregulation. This cytokine suppresses T cell proliferation via various mechanisms, such as by inducing the downregulation of cyclins and IL-2 and the upregulation of cyclin-dependent-kinases (CDKs) (29–31). TGF-β can also suppress the formation of effector CD4+ or CD8+ T cells by inhibiting the expression of the master Th1 and Th2 cell transcriptional regulators (T-bet and GATA-3, respectively) (32, 33) or the IL-12Rβ2 chain (34).

The contribution and importance of both cytokines, IL-10 and TGFβ, to the immunosuppressive activity of TR1 cells is exemplified by the fact that, blockade of these cytokines inhibits TR1 cell-mediated immunoregulation in various experimental settings, including pMHCII-NP-treated animals (2, 8).

An additional cytokine that has been implicated in TR1-mediated immunoregulation is IL-21. Whereas IL-10 is directly responsible for most of the regulatory properties of pMHCII-NP-induced TR1 cells, IL-21 contributes to sustaining IL-10 expression in TR1 cells and is directly responsible for TR1-induced Breg cell formation (8).

### Engagement of co-inhibitory and co-stimulatory molecules

Engagement of the TR1 cells’ co-inhibitory (i.e., LAG-3, CTLA-4, TIGIT or PD-1) and co-stimulatory receptors (i.e., ICOS) by the corresponding ligands on target cells, such as APCs, is also thought to play a role in their immunoregulatory activity. Indeed, all these molecules are upregulated on the TR1-like cells induced by pMHCII-NP therapy (9). Whereas engagement of co-inhibitory receptor ligands on APCs by the TR1 cells’ co-inhibitory receptors may contribute to the suppression of the APC’s function, engagement of co-stimulatory receptor ligands (along with cognate pMHCII) elicits the productive activation of the TR1 cells, leading to secretion of the TR1 cells’ immunoregulatory cytokines. In turn, these molecules have immunoregulatory effects on APCs and other downstream cellular targets.

LAG-3 negatively regulates T cell activity. Structurally similar to the CD4 co-receptor, LAG-3 recognizes MHCII molecules with higher affinity than CD4 (35). Recent evidence has shown that engagement of LAG-3 by stable pMHCII complexes transduces intracellular inhibitory signals to the T cell, without interfering with the recognition of these complexes by the T cells’ TCR or CD4 molecules (36). Although such a mechanism helps understand how LAG-3 upregulation by an effector T cell might suppress its activation, a T cell-intrinsic inhibitory role for LAG-3 on regulatory T cell activity/function (37) seems counter-intuitive, as it would suppress the Treg cell, suggesting the existence of alternative mechanisms. One possibility is that the interaction between LAG-3 on Treg cells and pMHCII on APCs exclusively results in suppression of the latter, perhaps by failing to transduce intracellular inhibitory signaling into the former. The finding that such interaction results in the inhibition of dendritic cell (DC) activation (38), supports this possibility.

CTLA-4, a member of the CD28 family, binds to the co-stimulatory ligands CD80/86 with higher affinity than CD28, inhibiting the activation of the latter. In addition, the CTLA-4–CD80/86 interaction promotes the dephosphorylation of CD3 and CD28 signalling intermediates through the Src homology region 2-containing protein tyrosine phosphatase-2 (SHP-2), promoting T cell inhibition (39). Although CTLA-4 is dispensable for peripheral Treg cell expansion, it is necessary for immunoregulatory activity (40). There is also evidence indicating that CTLA-4 (along with PD-1) plays an active role in the regulatory activity of TR1 cells (41). It therefore seems likely that the role of CTLA-4 expression on TR1/Treg cells is different than that of CTLA-4 upregulation by effector T cells, although this remains to be determined (42).

The PD-1 receptor binds PD-L1 or PD-L2, expressed predominantly on APCs. Upon interaction with PD-L2, PD-1 on effector T cells recruits SHP-1 and SHP-2 phosphatases, which in turn reduce T cell activation and induce Treg cell differentiation (43). On DCs, the PD1–PD-L2 interaction inhibits the expression of molecules associated with DC maturation such as CD80, CD86 or CD40 and induces IL-10 expression, thus promoting the induction of an immunosuppressive DC phenotype (44).

Although the intracellular domain of TIGIT contains an immunoreceptor tyrosine-based inhibitory motif (ITIM) capable of recruiting SHIP-1 and thus suppress T-cell (and NK cell) activation (45), binding of TIGIT to CD155 or CD112 on APCs (with high and low affinity, respectively), inhibits the engagement of the CD226 co-stimulator on T cells (46). In addition, this interaction induces a tolerogenic phenotype in DCs, by promoting IL-10 and suppressing IL-12 production (47).

Binding of the co-stimulator ICOS on TR1 cells to its ligand, ICOS-L, on B cells, DCs or macrophages (in the context of a cognate TCR-pMHCII interaction) promotes TR1 cell activation, leading to secretion of regulatory cytokines such IL-10 (48–50).

### Extracellular generation of adenosine

TR1 cells, including those arising in response to pMHCII-NP therapy (9) express ectonucleoside triphosphate diphosphohydrolase 1 (CD39) and ecto-5’-nucleotidase (CD73), which hydrolyze extracellular adenosine triphosphate (ATP) released during T cell activation (51). This leads to the generation of adenosine (52, 53), which binds to the G protein-coupled adenosine receptor A2 (A_2A_R). This interaction elicits a signalling cascade that suppresses effector T cell proliferation and cytokine production (54). On APCs, binding of adenosine to A_2A_R promotes IL-10 expression and inhibits both their maturation and their ability to secrete pro-inflammatory cytokines (55).

### Granzyme and perforin-mediated killing

Another mechanism that TR1 cells may use to regulate T cell activity involves the killing of cognate APCs (i.e., expressing the TR1 cells’ target pMHCII) via granzyme A and B and perforin (56, 57). By killing APCs, TR1 cells can thus suppres the activation of other T cell specificities and promote bystander immunoregulation. However, our work in mice treated with autoimmune disease-relevant pMHCII-NPs suggest that this mechanism is not always at play. For example, the antigen-specific TR1 cells emerging in these mice upon pMHCII-NP therapy did not significantly upregulate perforin and did not kill antigen-expressing or peptide-pulsed APCs (B cells or DCs) *in vivo* (8), unlike the case for the regulatory CD8+ T cells arising in pMHCI-NP-treated animals (58).

## Molecular and transcriptional regulation of TR1 cell specification

Extensive efforts over the last two decades have sought to define the molecular and transcriptional mechanisms orchestrating TR1 formation. Unlike the case for the FoxP3+ Treg cell subset, where expression of FoxP3 is central to the acquisition of its immunoregulatory properties, there is no known unique master transcriptional regulator of TR1 cell development. Notwithstanding this limitation, experimental evidence has implicated a number of cytokines, kinases and transcription factors in the generation of TR1 cells *in vitro*. Although TCR engagement in the presence of IL-10 appears to play a major role, other signals are also required.

There is evidence suggesting that superantigens (59, 60) and pMHCII multimers can induce the expression of IL-10 in CD4+ T cells (61–63). It has also been shown that high-avidity TCR–pMHCII interactions favour the production of IL-10 by T cells (64), affecting both the number of cells expressing IL-10 and the immunoregulatory properties of such cells (65). Molecularly, TCR activation leads to the engagement of intracellular signalling pathways that eventually activate the interferon regulatory factor 4 (IRF4) transcription factor via Ras or the inducible tyrosine kinase (ITK) kinases. IRF4 has been shown to promote *Il10* gene expression in different CD4+ T cell types, including Th2, Th1 (66), Tfh cells (67) and Tregs (68). Indeed, it has been reported that IRF4 contributes to the development of an IL-10-producing CD4+ T cell that co-expresses LAG-3 and CD49b (69). As noted below, IRF4 is absolutely required for pMHCII-NP-induced TR1 cell formation, albeit through a different mechanism (i.e., it is dissociated from its *Il10* transactivating function) (9). The transcription factor Eomes, which can promote *Il10* expression in T-bet-expressing cells (70), and the Th17 transcription factor Rorα, which can transactivate the *Il10* gene (71), might also be implicated in TR1 formation. However, pMHCII-NP-induced TR1 cells do not upregulate Eomes or Rorα, suggesting that neither of these transcription factors are required for TR1 cell specification.

Although productive TCR ligation is required for TR1 activation, TR1 cell genesis requires additional cues. Early studies by Groux and colleagues using both human and murine CD4+ T cells cultured in presence of IL-10 indicated that these culture conditions promoted the development of an anergic T cell population that included TR1-like cells (2), highlighting a prominent role for this cytokine in TR1 cell generation, at least *in vitro*. It was subsequently proposed that the IL-10 that contributes to TR1 cell generation *in vivo* derives from a tolerogenic DC population (72, 73). Indeed, a human DC population expressing high amounts of IL-10 has been identified (27). This DC population, named DC-10, can induce TR1 cells *ex vivo* with increased efficacy, as compared to other experimental approaches, and such cells have been used to generate and expand TR1 cells for use in clinical trials [Reviewed in (5)].

IL-27 has also been implicated in the generation of murine TR1 cells. IL-27 is an IL-12 family cytokine that is produced by activated APCs (74) and can induce IL-10 expression in murine T cells (75–77), especially in the presence of TGFβ, with which it synergizes (78). Binding of IL-27 to the IL-27 receptor (IL-27R) activates the STAT1 and STAT3 signalling pathways and promotes the expression of the transcription factors c-Maf and AhR, which cooperatively promote *Il10* and *Il21* expression (78, 79). STAT3-induced *Il10* expression also involves the upregulation of *Egr2* (encoding the Early Growth Response 2 transcription factor (EGR-2)) and EGR-2’s downstream target *Prdm1* (encoding the zinc finger-containing transcription factor Blimp-1) (80). Although Blimp-1 has been primarily implicated in plasma cell differentiation (81), it has also been shown to regulate *Il10* gene expression in T cells (82, 83). However, and notwithstanding the fact that the generation of terminally differentiated TR1 cells in response to pMHCII-NPs requires Blimp-1, this role is dissociated from Blimp-1’s *Il10* transactivating function (9) (see below). In fact, there is evidence suggesting that IL-27-induced TR1 cell formation does not require IL-27-induced IL-10 (84) and that IL-27 contributes to TR1 cell formation by inducing changes in chromatin accessibility via IRF1 and BAFT (85). Although IL-27 can induce the formation of IL-10-expressing T cells from naïve human CD4+ precursors (86), it remains to be determined whether these cells are *bona fide* TR1 cells.

Despite all these observations, largely if not exclusively generated *in vitro*, our *in vivo* work has demonstrated that IL-27 is not required for pMHCII-NP-induction of TR1-like cells (8). We have proposed that IL-27 and pMHCII-NPs lie upstream and downstream of the TR1 precursors (Tfh cells, see further below); whereas IL-27 would elicit both Tfh and TR1 cell formation from naïve precursors, pMHCII-NPs would just be able to promote the conversion of Tfh cells into TR1-like cells (9).

IL-21 is another cytokine that has been implicated in TR1 cell genesis. This cytokine, produced by antigen-stimulated CD4+ T cells and NKT cells, signals via the IL-21R, composed of the IL-21Rα chain and the common receptor γ_c_ chain (87), leading to activation of the STAT3 signalling pathway. The transcription factor c-Maf, upregulated by IL-27 among other stimuli, promotes IL-21 expression in TR1 cells (79, 88). In turn, IL-21 promotes the expression of *Il10* and *cMaf* expression in an autocrine manner (79, 89).

In addition to the molecules discussed above, other cytokines and transcription factors have been reported to contribute to IL-10 production by T cells and, consequently, may play a role in TR1 cell specification. For instance, IL-6, which signals through STAT1 and STAT3, can upregulate the transcription factors c-Maf, IRF4 or AhR (90), which are known to participate in *Il21 and/or l10* expression in different T cell types (66–69, 78, 79). In fact, IL-6, together with TGFβ, can induce IL-10 production in Th17 cells (91, 92). Type-1 Interferons have also been reported to promote IL-10 expression in CD4+ T cells (93–95) or TR1 cell development in anti-CD3 mAb/IL-10-treated mice (96).

The co-stimulator ICOS may also play an important role in TR1 cell specification, homeostasis or function, perhaps by promoting *cMaf* and *Il10/Il21* expression (88, 97).

In summary, research to date has provided valuable information regarding the transcriptional control of *Il10*, encoding the hallmark TR1 cytokine, but has not yet been able to define the key transcription factors, co-stimulators and cytokines that control TR1 cell development from their precursors. As summarized below, the recent identification of Tfh cells as precursors of TR1 cells *in vivo*, coupled with definition of the transcriptional changes that underlie this transdifferentiation process, offer a unique opportunity to carefully map the molecular events responsible for TR1 cell formation.

## Challenges hampering studies on the developmental biology of TR1 cells

Given the challenges associated with the lack of TR1 cell-specific markers and our inability to reliably identify this T cell subset *in vivo* until recently, it is unclear whether the TR1-like cells that have been described to arise *in vitro* and/or *in vivo* in response to various cues do so from a single or various precursors [reviewed in (98, 99)].

As noted above, both human and mouse T cells can be differentiated into TR1-like cells *in vitro*. *In vitro*-activated naïve T cells from either species can give rise to anergized IL-10-producing CD4+ T cells when cultured in the presence of exogenous IL-10 (2), DC-10 cells (100) or IL-27 (79, 101).

Several other lines of evidence have suggested that TR1 cells arise from memory T cell precursors. Repetitive administration of anti-CD3 monoclonal antibodies (mAb) to mice can induce TR1-like cell formation *in vivo* (102). *In vitro* stimulation of memory-like CD4^+^CD44^high^FoxP3^–^ T cells in the absence of polarizing cytokines can also elicit TR1-like cell specification (103). Likewise, extracellular matrix components have been reported to guide the formation of TR1-like cells from human memory CD4^+^ T cells *in vitro* (104), and others have shown that the precursors of TR1 cells are contained within the memory CD4^+^ T cell pool, in both humans and mice (105).

Th1 and Th2 cells have also been proposed as a source of TR1-like cells. *In vitro*, Th1 cells can be induced to express IL-10 when stimulated in the presence of CXCL12 (106), but such cells might have just been IL-10-producing Th1 cells rather than full-fledged TR1 cells. As noted earlier in this review, it has been suggested that chronic infection of mice with *Plasmodium* can trigger the differentiation of Th1 cells into TR1-like cells (15, 16), but the reported TR1-like cells did not appear to be *bona fide* TR1 cells. It has also been suggested that allergen-specific Th2 cells can be re-programmed into a TR1-like phenotype *in vitro* (107).

Intestinal Th17 cells can also give rise to anti-colitogenic TR1-like cells in response to anti-CD3 mAb treatment (108). Furthermore, IL-27 and IL-23 promote the up-regulation of Blimp-1 and can elicit the expression of a TR1-like phenotype in Th17 cells (109).

Collectively, the above observations suggest that TR1-like cells might arise from various T cell precursor types, but many of these studies did not use stringent criteria for definition of the TR1 cell state, or did not involve detailed transcriptional studies of the T cell pools used for experimentation or of their progeny; and when such studies were done/reported, the resulting T cell pools were transcriptionally heterogeneous. As a result, it is not possible to unambiguously assign or exclude a specific cell type as a TR1 cell precursor on the basis of these studies.

## Tfh cells as a source of TR1 cells

**Figure 2:**
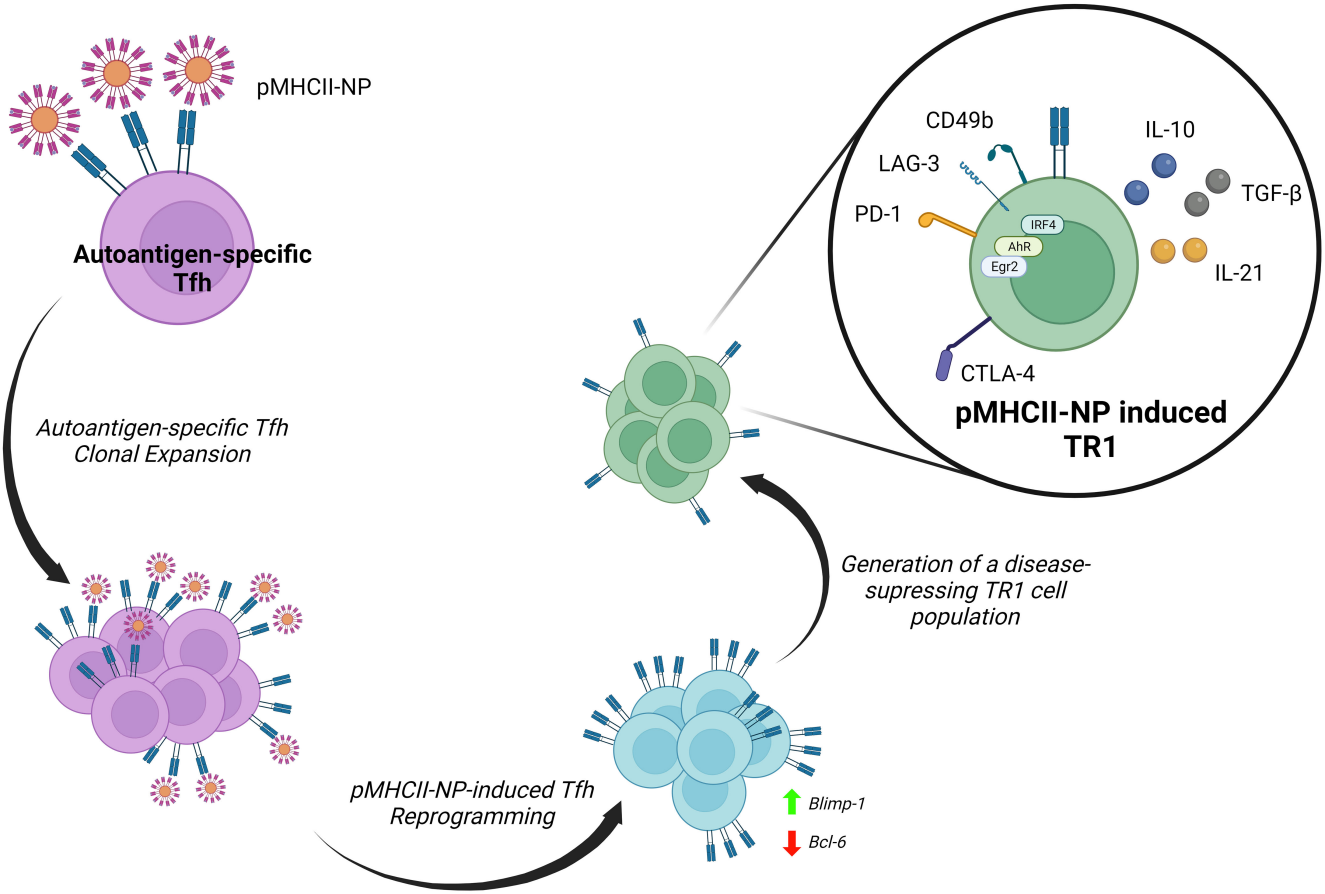
Reprogramming of Tfh cells into TR1 progeny by pMHCII-NPs. Upon binding to cognate TCRs, autoimmune disease relevant pMHCII-NPs selectively trigger the clonal expansion of Tfh cells and their immediate transdifferentiation into transitional TR1-like cells and terminally differentiated TR1 cells. pMHCII-NP encounters trigger the downregulation of master transcriptional regulators of Tfh cells, such as Bcl-6, and the upregulation of Blimp-1. Blimp-1 expression is a *sine-qua-non* requirement for the conversion of transitional TR1-like cells into the terminally differentiated, immunoregulatory TR1 cell subset (9).

We have taken advantage of the large pools of antigen-specific TR1 cells that arise *in vivo* in various animal models of autoimmunity upon systemic delivery of nanoparticles (NPs) coated with disease-relevant pMHCII molecules (8, 21–23), to carefully map the transcriptional events leading to TR1 cell formation *in vivo* (9, 10) (**Figure 2**). Early work established that pMHCII-NPs functioned by re-programming cognate antigen-experienced (i.e. memory) CD4^+^ T-cells of unknown identity (excluding a role for naïve T-cells) (8, 23).

The robust and prolongued TCR signaling events that result from sustained assembly of TCR microclusters by pMHCII-NPs on autoantigen-experienced T cells leads to the expression of known TR1-like cell markers, including IL-10, IL-21, c-Maf, LAG-3, CD49b, CTLA-4, PD-1, TIGIT, CCR5, CXCR3, ICOS and OX-40, among others, in a manner that does not require APCs or IL-27 (9). In addition to *Maf*, these TR1 cells upregulate the transcription factor coding genes *Ahr, Egr2, Irf4, Nfil3, Prdm1 and Tbx21* (9), all involved in IL-10 expression (79, 110). In addition, these TR1 cells upregulate three other transcription factors that have been previously implicated in the development, maintenance or function of IL-10-expressing Treg cells (*Bhlhe40*, *Runx2* and *Vdr*) (9). Whereas the IL-10 produced by these antigen-specific TR1 cells is the direct mediator of some of their immunoregulatory properties, IL-21 contributes to the homeostatic regulation of this T-cell subset and plays a critical role in TR1-induced Breg cell formation (8). This ability of pMHCII-NPs to elicit the formation of large pools of antigen-specific TR1-like cells afforded us a unique opportunity to explore their developmental biology. This work has demonstrated that pMHCII-NP-induced TR1 cells derive from cognate Tfh cells and do so in a Blimp-1-dependent manner (9, 10) (**Figure 2**).

Initial work indicated that the cognate TR1-like cell pools arising in response to pMHCII-NPs expressed a transcriptional program that shared significant features with Tfh cells, raising the possibility that the latter might function as a source of the former. Subsequent single cell RNA sequencing (scRNAseq) and mass cytometry studies demonstrated that these antigen-specific TR1-like cell pools harboured a cognate Tfh-like cell subcluster, in addition to its TR1-like cell countepart. Importantly, studies of the TCR repertoires of these two cell sub-clusters indicated that they consistently harbored identical clonotypes, thus demonstrating that they were developmentally related (9, 10). This was substantiated with the use of different pMHCII-NP types in different genetic backgrounds and models of autoimmunity (9, 10).

This was further documented by demonstrating that pMHCII-NPs could elicit cognate TR1 cell formation in immunocompromised hosts transfused with purified Tfh cells, and that these compounds lacked pharmacodynamic activity in mice unable to generate Tfh cells (9). Most importantly, T cell-specific deletion of *Prdm1* (encoding Blimp-1) revealed that the Tfh-to-TR1 cell conversion evolves through a transitional (TR1-like) subset, and that expression of this transcription factor in these transitional T cells is a *sine qua non* requirement for full-fledged acquisition of the TR1 transcriptional profile and regulatory function (9). Thus, while specific deletion of *Bcl6* or *Irf4* in T-cells blunted pMHCII-NP-induced cognate CD4+ T-cell expansion and downstream TR1 generation, deletion of *Prdm1* enabled the former but completely abrogated the latter (9).

It could be argued that pMHCII-NP-induced TR1 cells are T-follicular regulatory (TFR) cells, which negatively regulate the germinal center (GC) reaction (111). However, unlike TR1 cells, TFR cells express CXCR5 (but not CCR5), Bcl-6, FoxP3 and CD25, and arise from natural FoxP3+ Treg cell precursors in a Blimp-1-independent manner (9).

Together, the data summarized above conclusively demonstrate that murine TR1 cells can arise from Tfh cells in a Blimp-1-dependent manner. Interestingly, a pool of thymus-derived self-reactive CD4+ T cells that adopt numerous hallmarks of Tfh cell identity in the periphery has been recently discovered (112). This finding raises the possibility that these cells might function as a source of a negative feedback regulatory loop (i.e., formation of autoreactive TR1 cells) to suppress autoimmunity.

## Concluding statement

Since the discovery of TR1-like cells more than 25 years ago, the last decade has witnessed steady improvements in our ability to identify and phenotype this previously enigmatic CD4+ T cell  subset. While knowledge gaps persist, we have gained detailed new insights into these cells’ transcriptional make-up, mechanisms of action and lineage origin. Further research into the different topics reviewed in this article, as well as other aspects of the TR1 cell biology will undoubtedly help in the translational application of TR1 cells as a therapeutic approach for immune-mediated diseases.

## Author contributions

PS: Conceptualization, Funding acquisition, Investigation, Project administration, Resources, Supervision, Writing – review and editing. EA: Investigation, Writing – original draft.

## References

[1] Roncarolo MG Yssel H Touraine JL Betuel H De Vries JE Spits H. Autoreactive T cell clones specific for class I and class II HLA antigens isolated from a human chimera. J Exp Med (1988) 167:1523–34. doi: 10.1084/jem.167.5.1523 PMC2188931 3284961

[2] Groux H O'Garra A Bigler M Rouleau M Antonenko S De Vries JE. A CD4+T-cell subset inhibits antigen-specific T-cell responses and prevents colitis. Nature (1997) 389:737–42. doi: 10.1038/39614 9338786

[3] Gregori S Roncarolo MG Bacchetta R. Methods for *in vitro* generation of human type 1 regulatory T cells. Methods Mol Biol (2011) 677:31–46. doi: 10.1007/978-1-60761-869-0_3 20941601

[4] Desreumaux P Foussat A Allez M Beaugerie L Hébuterne X Bouhnik Y. Safety and efficacy of antigen-specific regulatory T-cell therapy for patients with refractory Crohn's disease. Gastroenterology (2012) 143:1207–17. doi: 10.1053/j.gastro.2012.07.116 22885333

[5] Gregori S Roncarolo MG. Engineered T regulatory type 1 cells for clinical application. Front Immunol (2018) 9:233–3. doi: 10.3389/fimmu.2018.00233 PMC5818395 29497421

[6] Bacchetta R Lucarelli B Sartirana C Gregori S Lupo Stanghellini MT Miqueu P. Immunological outcome in haploidentical-HSC transplanted patients treated with IL-10-anergized donor T cells. Front Immunol (2014) 5. doi: 10.3389/fimmu.2014.00016 PMC3907718 24550909

[7] Brockmann L Soukou S Steglich B Czarnewski P Zhao L Wende S. Molecular and functional heterogeneity of IL-10-producing CD4+ T cells. Nat Commun (2018) 9:5417. doi: 10.1038/s41467-018-07581-4 30575716 PMC6303294

[8] Clemente-Casares X Blanco J Ambalavanan P Yamanouchi J Singha S Fandos C. Expanding antigen-specific regulatory networks to treat autoimmunity. Nature (2016) 530:434–40. doi: 10.1038/nature16962 26886799

[9] Solé P YJ Garnica J Myn Uddin M Clarke R Moro J Garabatos N. A T follicular helper cell origin for T regulatory type 1 cells. Cell Mol Immunol (2023) 20:489–511. doi: 10.1038/s41423-023-00989-z 36973489 PMC10202951

[10] Solé P Parras D Yamanouchi J Garnica J Garabatos N Moro J. Transcriptional re-programming of insulin B-chain epitope-specific T-follicular helper cells into anti-diabetogenic T-regulatory type-1 cells. Front Immunol (2023) 14:1744–4. doi: 10.3389/fimmu.2023.1177722 PMC10154693 37153608

[11] Bacchetta R Bigler M Touraine JL Parkman R Tovo PA Abrams J. High levels of interleukin 10 production in *vivo* are associated with tolerance in SCID patients transplanted with HLA mismatched hematopoietic stem cells. J Exp Med (1994) 179:493–502. doi: 10.1084/jem.179.2.493 7905018 PMC2191349

[12] Cope A Le Friec G Cardone J Kemper C. The Th1 life cycle: molecular control of IFN-g to IL-10 switching. Trends Immunol (2011) 32:278–86. doi: 10.1016/j.it.2011.03.010 21531623

[13] Gabrysova L Nicolson K Streeter H Verhagen J Sabatos-Peyton CA Morgan D. Negative feedback control of the autoimmune response through antigen-induced differentiation of IL-10-secreting Th1 cells. J Exp Med (2009) 206:1755–67. doi: 10.1084/jem.20082118 PMC2722173 19635862

[14] Atlin J Goodknow C Cook M. IL-10+ CTLA-4+ Th2 Inhibitory Cells Form in aFoxp3-Independent, IL-2–Dependent Manner from Th2 Effectors during Chronic Inflammation. J Immunol (2012) 188(11):5478–88. doi: 10.4049/jimmunol.1102994 22547705

[15] Lönnberg T Svensson V James KR Fernandez-Ruiz D Sebina I Montandon R. Single-cell RNA-seq and computational analysis using temporal mixture modelling resolves Th1/Tfh fate bifurcation in malaria. Sci Immunol (2017) 2(9):eaal2192. doi: 10.1126/sciimmunol.aal2192 28345074 PMC5365145

[16] Soon MSF Lee HJ Engel JA Straube J Thomas BS Pernold CPS. Transcriptome dynamics of CD4(+) T cells during malaria maps gradual transit from effector to memory. Nat Immunol (2020) 21:1597–610. doi: 10.1038/s41590-020-0800-8 33046889

[17] Freeborn RA Strubbe S Grazia Roncarolo M. Type 1 regulatory T cell-mediated tolerance in health and disease. Front Immunol (2022) 13. doi: 10.3389/fimmu.2022.1032575 PMC9650496 36389662

[18] Gagliani N Magnani CF Huber S Gianolini ME Pala M Licona-Limon P. Coexpression of CD49b and LAG-3 identifies human and mouse T regulatory type 1 cells. Nat Med (2013) 19:739–46. doi: 10.1038/nm.3179 23624599

[19] Alfen J Larghi P Facciotti F Gagliani N Bosotti R Paroni M. Intestinal IFN-g–producing type 1 regulatory T cells coexpress CCR5 and programmed cell death protein 1 and downregulate IL-10 in the inflamed guts of patients with inflammatory bowel disease. J Allergy Clin Immunol (2018) 142:P1537–1547.E8. doi: 10.1016/j.jaci.2017.12.984 29369775

[20] Roncarolo MG Gregori S Bacchetta R Battaglia M Gagliani N. The biology of T regulatory type 1 cells and their therapeutic application in immune-mediated diseases. Immunity (2018) 49:1004–19. doi: 10.1016/j.immuni.2018.12.001 30566879

[21] Umeshappa CS Mbongue J Singha S Mohapatra S Yamanouchi J Lee JA. Ubiquitous antigen-specific T regulatory type 1 cells variably suppress hepatic and extrahepatic autoimmunity. J Clin Invest (2020) 130:1823–9. doi: 10.1172/JCI130670 PMC7108901 32125290

[22] Umeshappa CS Singha S Blanco J Shao K Nanjundappa RH Yamanouchi J. Suppression of a broad spectrum of liver autoimmune pathologies by single peptide-MHC-based nanomedicines. Nat Commun (2019) 10:2150. doi: 10.1038/s41467-019-09893-5 31089130 PMC6517389

[23] Singha S Shao K Yang Y Clemente-Casares X Solé P Clemente A. Peptide-MHC-based nanomedicines for autoimmunity function as T-cell receptor microclustering devices. Nat Nanotechnol (2017) 12:701–10. doi: 10.1038/nnano.2017.56 28436959

[24] Taga K Tosato G. IL-10 inhibits human T cell proliferation and IL-2 production. J Immunol (1992) 148:1143–8. doi: 10.4049/jimmunol.148.4.1143 1737931

[25] Murray P. The primary mechanism of the IL-10-regulated antiinflammatory response is to selectively inhibit transcription. Proc Natl Acad Sci United States America (2005) 102:8686–91. doi: 10.1073/pnas.0500419102 PMC1150817 15937121

[26] Moore K de Waal Malefyt R Coffman R O’Garra A. Interleukin-10 and the interleukin-10 receptor. Annu Rev Immunol (2001) 19:683–765. doi: 10.1146/annurev.immunol.19.1.683 11244051

[27] Gregori S Tomasoni D Pacciani V Scirpoli M Battaglia M Magnani CF. Differentiation of type 1 T regulatory cells ( Tr1 ) by tolerogenic DC-10 requires the IL-10 – dependent ILT4 / HLA-G pathway. BLOOD (2010) 116:935–44. doi: 10.1182/blood-2009-07-234872 20448110

[28] Satoguina J Weyand E Larbi J Hoerauf A. T regulatory-1 cells induce igG4 production by B cells: role of IL-10. J Immunol (2005) 174:4718–26. doi: 10.4049/jimmunol.174.8.4718 15814696

[29] Datto MB Li Y Panus JF Howe DJ Xiong Y Wang XF. Transforming growth factor beta induces the cyclin-dependent kinase inhibitor p21 through a p53-independent mechanism. Proc Natl Acad Sci U.S.A. (1995) 92:5545–9. doi: 10.1073/pnas.92.12.5545 PMC41732 7777546

[30] Hannon GJ Beach D. p15INK4B is a potential effector of TGF-beta-induced cell cycle arrest. Nature (1994) 371:257–61. doi: 10.1038/371257a0 8078588

[31] Brabletz T Pfeuffer I Schorr E Siebelt F Wirth T Serfling E. Transforming growth factor beta and cyclosporin A inhibit the inducible activity of the interleukin-2 gene in T cells through a noncanonical octamer-binding site. Mol Cell Biol (1993) 13:1155–62. doi: 10.1128/MCB.13.2.1155 PMC359000 8423782

[32] Gorelik L Fields PE Flavell RA. Cutting edge: TGF-beta inhibits Th type 2 development through inhibition of GATA-3 expression. J Immunol (2000) 165:4773–7. doi: 10.4049/jimmunol.165.9.4773 11045997

[33] Park IK Shultz LD Letterio JJ Gorham JD. TGF-beta1 inhibits T-bet induction by IFN-gamma in murine CD4+ T cells through the protein tyrosine phosphatase Src homology region 2 domain-containing phosphatase-1. J Immunol (2005) 175:5666–74. doi: 10.4049/jimmunol.175.9.5666 16237056

[34] Gorham JD Güler ML Fenoglio D Gubler U Murphy KM. Low dose TGF-beta attenuates IL-12 responsiveness in murine Th cells. J Immunol (1998) 161:1664–70. doi: 10.4049/jimmunol.161.4.1664 9712029

[35] Workman C Rice D Dugger K Kurschner C Vignali D. Phenotypic analysis of the murine CD4-related glycoprotein, CD223 (LAG-3). Eur J Immunol (2002) 32:2253–63. doi: 10.1002/1521-4141(200208)32:8<2255::AID-IMMU2255>3.0.CO;2-A 12209638

[36] Maruhashi T Okazaki I Sugiura D Takahashi S Maeda T Shimizu K. LAG-3 inhibits the activation of CD4+ T cells that recognize stable pMHCII through its conformation-dependent recognition of pMHCII. Nat Immunol (2018) 19:1415–26. doi: 10.1038/s41590-018-0217-9 30349037

[37] Ruffo E Wu RC Bruno TC Workman CJ Vignali DAA. Lymphocyte-activation gene 3 (LAG3): The next immune checkpoint receptor. Semin Immunol (2019) 42:101305. doi: 10.1016/j.smim.2019.101305 31604537 PMC6920665

[38] Liang B Workman C Lee J Chew C Dale B Colonna L. Regulatory T cells inhibit dendritic cells by lymphocyte activation gene-3 engagement of MHC class II. J Immunol (2008) 180:5916–26. doi: 10.4049/jimmunol.180.9.5916 18424711

[39] Lee K Chouang E Griffin M Khattri R Hong DK Zhang W. Molecular basis of T cell inactivation by CTLA-4. Science (1998) 284:2263–6. doi: 10.1126/science.282.5397.2263 9856951

[40] Schmidt EM Wang CJ Ryan GA Clough LE Qureshi OS Goodall M. Ctla-4 controls regulatory T cell peripheral homeostasis and is required for suppression of pancreatic islet autoimmunity. J Immunol (2009) 182:274–82. doi: 10.4049/jimmunol.182.1.274 19109158

[41] Chen PP Cepika AM Agarwal-Hashmi R Saini G Uyeda MJ Louis DM. Alloantigen-specific type 1 regulatory T cells suppress through CTLA-4 and PD-1 pathways and persist long-term in patients. Sci Transl Med (2021) 13:eabf5264. doi: 10.1126/scitranslmed.abf5264 34705520 PMC9451143

[42] De Sousa Linhares A Leitner J Grabmeier-Pfistershammer K Steinberger P. Not all immune checkpoints are created equal. Front Immunol (2018) 9:1909. doi: 10.3389/fimmu.2018.01909 30233564 PMC6127213

[43] Sharpe AH Pauken KE. The diverse functions of the PD1 inhibitory pathway. Nat Rev Immunol (2017) 18:153–67. doi: 10.1038/nri.2017.108 28990585

[44] Sharpe AH Wherry E Ahmed R Freeman G. The function of programmed cell death 1 and its ligands in regulating autoimmunity and infection. Nat Immunol (2007) 8:239–45. doi: 10.1038/ni1443 17304234

[45] Liu S Zhang H Li M Hu D Li C Ge B. Recruitment of Grb2 and SHIP1 by the ITT-like motif of TIGIT suppresses granule polarization and cytotoxicity of NK cells. Cell Death Differentiation (2013) 20:456–64. doi: 10.1038/cdd.2012.141 PMC3569986 23154388

[46] Zhang B Zhao W Li H Chen Y Tian H Li L. Immunoreceptor TIGIT inhibits the cytotoxicity of human cytokine-induced killer cells by interacting with CD155. Cancer Immunol Immunother (2016) 65:305–14. doi: 10.1007/s00262-016-1799-4 PMC11029225 26842126

[47] Yu X Harden K Gonzalez LC Francesco M Chiang E Irving B. The surface protein TIGIT suppresses T cell activation by promoting the generation of mature immunoregulatory dendritic cells. Nat Immunol (2009) 10:48–57. doi: 10.1038/ni.1674 19011627

[48] Dong C Juedes AE Temann U-E Shresta S Allison JP Ruddle NH. ICOS co-stimulatory receptor is essential for T-cell activation and function. Nature (2001) 409:97–101. doi: 10.1038/35051100 11343121

[49] Hutloff A Dittrich AM Beier KC Eljaschewitsch B Kraft R Anagnostopoulos I. ICOS is an inducible T-cell co-stimulator structurally and functionally related to CD28. Nature (1999) 397:263–6. doi: 10.1038/16717 9930702

[50] Witsch EJ Peiser M Hutloff A Büchner K Dorner BG Jonuleit H. ICOS and CD28 reversely regulate IL-10 on re-activation of human effector T cells with mature dendritic cells. Eur J Immunol (2002) 32:2680–6. doi: 10.1002/1521-4141(200209)32:9<2680::AID-IMMU2680>3.0.CO;2-6 12207353

[51] Junger WG. Immune cell regulation by autocrine purinergic signalling. Nat Rev Immunol (2011) 11:201–12. doi: 10.1038/nri2938 PMC4209705 21331080

[52] Colgan SP Eltzschig HK Eckle T Thompson LF. Physiological roles for ecto-5’-nucleotidase (CD73). Purinergic Signalling (2006) 2:351–60. doi: 10.1007/s11302-005-5302-5 PMC2254482 18404475

[53] Allard B Longhi MS Robson SC Stagg J. The ectonucleotidases CD39 and CD73: Novel checkpoint inhibitor targets. Immunol Rev (2017) 276:121–44. doi: 10.1111/imr.12528 PMC5338647 28258700

[54] Mandapathil M Lang S Gorelik E Whiteside TL. Isolation of functional human regulatory T cells (Treg) from the peripheral blood based on the CD39 expression. J Immunol Methods (2009) 346:55–63. doi: 10.1016/j.jim.2009.05.004 19450601 PMC2703678

[55] Ben Addi A Lefort A Hua X Libert F Communi D Ledent C. Modulation of murine dendritic cell function by adenine nucleotides and adenosine: Involvement of the A2B receptor. Eur J Immunol (2008) 38:1610–20. doi: 10.1002/eji.200737781 18465770

[56] Grossman WJ Verbsky JW Tollefsen BL Kemper C Atkinson JP Ley TJ. Differential expression of granzymes A and B in human cytotoxic lymphocyte subsets and T regulatory cells. Blood (2004) 104:2840–8. doi: 10.1182/blood-2004-03-0859 15238416

[57] Magnani CF Alberigo G Bacchetta R Serafini G Andreani M Roncarolo MG. Killing of myeloid APCs via HLA class I, CD2 and CD226 defines a novel mechanism of suppression by human Tr1 cells. Eur J Immunol (2011) 41:1652–62. doi: 10.1002/eji.201041120 PMC3116154 21469116

[58] Tsai S Shameli A Yamanouchi J Clemente-Casares X Wang J Serra P. Reversal of autoimmunity by boosting memory-like autoregulatory T cells. Immunity (2010) 32:568–80. doi: 10.1016/j.immuni.2010.03.015 20381385

[59] Grundström S Cederbom L Sundstedt A Scheipers P Ivars F. Superantigen-induced regulatory T cells display different suppressive functions in the presence or absence of natural CD4+CD25+ regulatory T cells in vivo. J Immunol (2003) 170:5008–17. doi: 10.4049/jimmunol.170.10.5008 12734345

[60] Taylor AL Llewelyn MJ. Superantigen-induced proliferation of human CD4+CD25- T cells is followed by a switch to a functional regulatory phenotype. J Immunol (2010) 185:6591–8. doi: 10.4049/jimmunol.1002416 21048104

[61] Casares S Hurtado A McEvoy RC Sarukhan A von Boehmer H Brumeanu TD. Down-regulation of diabetogenic CD4+ T cells by a soluble dimeric peptide-MHC class II chimera. Nat Immunol (2002) 3:383–91. doi: 10.1038/ni770 11862219

[62] Masteller EL Warner MR Ferlin W Judkowski V Wilson D Glaichenhaus N. Peptide-MHC class II dimers as therapeutics to modulate antigen-specific T cell responses in autoimmune diabetes. J Immunol (2003) 171:5587–95. doi: 10.4049/jimmunol.171.10.5587 14607967

[63] Li L Yi Z Wang B Tisch R. Suppression of ongoing T cell-mediated autoimmunity by peptide-MHC class II dimer vaccination. J Immunol (2009) 183:4809–16. doi: 10.4049/jimmunol.0901616 PMC5444462 19752238

[64] Metzler B Wraith DC. Inhibition of experimental autoimmune encephalomyelitis by inhalation but not oral administration of the encephalitogenic peptide: influence of MHC binding affinity. Int Immunol (1993) 5:1159–65. doi: 10.1093/intimm/5.9.1159 7694644

[65] Gabrysová L Wraith DC. Antigenic strength controls the generation of antigen-specific IL-10-secreting T regulatory cells. Eur J Immunol (2010) 40:1386–95. doi: 10.1002/eji.200940151 PMC3466465 20162554

[66] Ahyi AN Chang HC Dent AL Nutt SL Kaplan MH. IFN regulatory factor 4 regulates the expression of a subset of Th2 cytokines. J Immunol (2009) 183:1598–606. doi: 10.4049/jimmunol.0803302 PMC2734910 19592658

[67] Kwon H Thierry-Mieg D Thierry-Mieg J Kim HP Oh J Tunyaplin C. Analysis of interleukin-21-induced Prdm1 gene regulation reveals functional cooperation of STAT3 and IRF4 transcription factors. Immunity (2009) 31:941–52. doi: 10.1016/j.immuni.2009.10.008 PMC3272079 20064451

[68] Cretney E Xin A Shi W Minnich M Masson F Miasari M. The transcription factors Blimp-1 and IRF4 jointly control the differentiation and function of effector regulatory T cells. Nat Immunol (2011) 12:304–11. doi: 10.1038/ni.2006 21378976

[69] Huang W Solouki S Koylass N Zheng SG August A. ITK signalling via the Ras/IRF4 pathway regulates the development and function of Tr1 cells. Nat Commun (2017) 8:15871. doi: 10.1038/ncomms15871 28635957 PMC5482062

[70] Zhang P Lee JS Gartlan KH Schuste IS Comerford I Varelias A. Eomesodermin promotes the development of type 1 regulatory T (TR1) cells. Sci Immunol (2017) 2(10):eaah7152. doi: 10.1126/sciimmunol.aah7152 28738016 PMC5714294

[71] Farez MF Mascanfroni ID Méndez-Huergo SP Yeste A Murugaiyan G Garo LP. Melatonin contributes to the seasonality of multiple sclerosis relapses. Cell (2015) 162:1338–52. doi: 10.1016/j.cell.2015.08.025 PMC4570563 26359987

[72] Levings MK Gregori S Tresoldi E Cazzaniga S Bonini C Roncarolo MG. Differentiation of Tr1 cells by immature dendritic cells requires IL-10 but not CD25+CD4+ Tr cells. Blood (2005) 105:1162–9. doi: 10.1182/blood-2004-03-1211 15479730

[73] Shiokawa A Tanabe K Tsuji NM Sato R Hachimura S. IL-10 and IL-27 producing dendritic cells capable of enhancing IL-10 production of T cells are induced in oral tolerance. Immunol Lett (2009) 125:7–14. doi: 10.1016/j.imlet.2009.05.002 19446579

[74] Pflanz S Timans JC Cheung J Rosales R Kanzler H Gilbert J. IL-27, a heterodimeric cytokine composed of EBI3 and p28 protein, induces proliferation of naive CD4+ T cells. Immunity (2002) 16:779–90. doi: 10.1016/S1074-7613(02)00324-2 12121660

[75] Awasthi A Carrier Y Peron JPS Bettelli E Kamanaka M Flavell RA. A dominant function for interleukin 27 in generating interleukin 10–producing anti-inflammatory T cells. Nat Immunol (2007) 8:1380–9. doi: 10.1038/ni1541 17994022

[76] Batten M Kljavin NM Li J Walter MJ de Sauvage FJ Ghilardi N. Cutting edge: IL-27 is a potent inducer of IL-10 but not FoxP3 in murine T cells. J Immunol (2008) 180:2752–6. doi: 10.4049/jimmunol.180.5.2752 18292493

[77] Fitzgerald DC Zhang GX El-Behi M Fonseca-Kelly Z Li H Yu S. Suppression of autoimmune inflammation of the central nervous system by interleukin 10 secreted by interleukin 27-stimulated T cells. Nat Immunol (2007) 8:1372–9. doi: 10.1038/ni1540 17994023

[78] Quintana FJ Basso AS Iglesias AH Korn T Farez MF Bettelli E. Control of Treg and TH17 cell differentiation by the aryl hydrocarbon receptor. Nature (2008) 453:65–71. doi: 10.1038/nature06880 18362915

[79] Pot C Jin H Awasthi A Liu SM Lai C-Y Madan R. Cutting edge: IL-27 induces the transcription factor c-Maf, cytokine IL-21, and the costimulatory receptor ICOS that coordinately act together to promote differentiation of IL-10-producing Tr1 cells. J Immunol (2009) 183:797–801. doi: 10.4049/jimmunol.0901233 19570826 PMC2768608

[80] Iwasaki Y Fujio K Okamura T Yanai A Sumitomo S Shoda H. Egr-2 transcription factor is required for Blimp-1-mediated IL-10 production in IL-27-stimulated CD4+ T cells. Eur J Immunol (2013) 43:1063–73. doi: 10.1002/eji.201242942 23349024

[81] Crotty S Johnston RJ Schoenberger SP. Effectors and memories: Bcl-6 and Blimp-1 in T and B lymphocyte differentiation. Nat Immunol (2010) 11:114–20. doi: 10.1038/ni.1837 PMC2864556 20084069

[82] Neumann C Heinrich F Neumann K Junghans V Mashreghi MF Ahlers J. Role of blimp-1 in programing th effector cells into IL-10 producers. J Exp Med (2014) 211:1807–19. doi: 10.1084/jem.20131548 PMC4144744 25073792

[83] Montes de Oca M Kumar R de Labastida Rivera F Amante FH Sheel M Faleiro RJ. Blimp-1-dependent IL-10 production by tr1 cells regulates TNF-mediated tissue pathology. PloS Pathog (2016) 12:e1005398. doi: 10.1371/journal.ppat.1005398 26765224 PMC4713066

[84] Maynard CL Harrington LE Janowski KM Oliver JR Zindl CL Rudensky AY. Regulatory T cells expressing interleukin 10 develop from Foxp3+ and Foxp3– precursor cells in the absence of interleukin 10. Nat Immunol (2007) 8:931–41. doi: 10.1038/ni1504 17694059

[85] Karwacz K Miraldi ER Pokrovskii M Madi A Yosef N Wortman I. Critical role of IRF1 and BATF in forming chromatin landscape during type 1 regulatory cell differentiation. Nat Immunol (2017) 18:412–21. doi: 10.1038/ni.3683 PMC5901650 28166218

[86] Murugaiyan G Mittal A Lopez-Diego R Maier LM Anderson DE Weiner HL. IL-27 is a key regulator of IL-10 and IL-17 production by human CD4+ T cells. J Immunol (2009) 183:2435–43. doi: 10.4049/jimmunol.0900568 PMC2904948 19625647

[87] Parrish-Novak J Dillon SR Nelson A Hammond A Sprecher C Gross JA. Interleukin 21 and its receptor are involved in NK cell expansion and regulation of lymphocyte function. Nature (2000) 408:57–63. doi: 10.1038/35040504 11081504

[88] Bauquet AT Jin H Paterson AM Mitsdoerffer M Ho I-C Sharpe AH. Costimulatory molecule ICOS plays a critical role in the development of TH-17 and follicular T-helper cells by regulating c-Maf expression and IL-21 production. Nat Immunol (2009) 10:167–75. doi: 10.1038/ni.1690 PMC2742982 19098919

[89] Spolski R Kim H-P Zhu W Levy DE Leonard WJ. IL-21 mediates suppressive effects via its induction of IL-10. J Immunol (2009) 182:2859–67. doi: 10.4049/jimmunol.0802978 PMC2756221 19234181

[90] Jin J-O Han X Yu Q. Interleukin-6 induces the generation of IL-10-producing Tr1 cells and suppresses autoimmune tissue inflammation. J Autoimmun (2013) 40:28–44. doi: 10.1016/j.jaut.2012.07.009 22921334 PMC3524403

[91] McGeachy MJ Bak-Jensen KS Chen Y Tato CM Blumenschein W McClanahan T. TGF-band IL-6 drive the production of IL-17 andIL-10 by T cells and restrain TH-17 cell-mediated pathology. Nat Immunol (2007) 8:1390–7. doi: 10.1038/ni1539 17994024

[92] Stumhofer JS Silver JS Laurence A Porrett PM Harris TH Turka LA. Interleukins 27 and 6 induce STAT3-mediated T cell production of interleukin 10. Nat Immunol (2007) 8:1363–71. doi: 10.1038/ni1537 17994025

[93] Aman MJ Tretter T Eisenbeis I Bug G Decker T Aulitzky WE. Interferon-alpha stimulates production of interleukin-10 in activated CD4+ T cells and monocytes. Blood (1996) 87:4731–6. doi: 10.1182/blood.V87.11.4731.bloodjournal87114731 8639843

[94] Garcia CA Benakanakere MR Alard P Kosiewicz MM Kinane DF Martin M. Antigenic experience dictates functional role of glycogen synthase kinase-3 in human CD4+ T cell responses. J Immunol (2008) 181:8363–71. doi: 10.4049/jimmunol.181.12.8363 PMC2849970 19050253

[95] McRae BL Semnani RT Hayes MP van Seventer GA. Type I IFNs inhibit human dendritic cell IL-12 production and Th1 cell development. J Immunol (1998) 160:4298–304. doi: 10.4049/jimmunol.160.9.4298 9574532

[96] Levings MK Sangregorio R Galbiati F Squadrone S de Waal Malefyt R Roncarolo M-G. IFN-α and IL-10 induce the differentiation of human type 1 T regulatory cells. J Immunol (2001) 166:5530–9. doi: 10.4049/jimmunol.166.9.5530 11313392

[97] Nurieva RI Duong J Kishikawa H Dianzani U Rojo JM Ho I-C. Transcriptional regulation of th2 differentiation by inducible costimulator. Immunity (2003) 18:801–11. doi: 10.1016/S1074-7613(03)00144-4 12818161

[98] Serra P Santamaria P. Antigen-specific therapeutic approaches for autoimmunity. Nat Biotechnol (2019) 37:238–51. doi: 10.1038/s41587-019-0015-4 30804535

[99] Solé P Santamaria P. Re-programming autoreactive T cells into T-regulatory type 1 cells for the treatment of autoimmunity. Front Immunol (2021) 12. doi: 10.3389/FIMMU.2023.1177722 PMC8320845 34335585

[100] Bacchetta R Gregori S Serafini G Sartirana C Schulz U Zino E. Molecular and functional characterization of allogantigen-specific anergic T cells suitable for cell therapy. Haematologica (2010) 95:2134–43. doi: 10.3324/haematol.2010.025825 PMC2995573 20713457

[101] Apetoh L Quintana FJ Pot C Joller N Xiao S Kumar D. The aryl hydrocarbon receptor interacts with c-Maf to promote the differentiation of type 1 regulatory T cells induced by IL-27. Nat Immunol (2010) 11:854–61. doi: 10.1038/ni.1912 PMC2940320 20676095

[102] Kamanaka M Kim ST Wan YY Sutterwala FS Lara-Tejero M Galán JE. Expression of interleukin-10 in intestinal lymphocytes detected by an interleukin-10 reporter knockin tiger mouse. Immunity (2006) 25:941–52. doi: 10.1016/j.immuni.2006.09.013 17137799

[103] Yao Y Vent-Schmidt J McGeough MD Wong M Hoffman HM Steiner TS. Tr1 Cells, but Not Foxp3+ Regulatory T Cells, Suppress NLRP3 Inflammasome Activation via an IL-10-Dependent Mechanism. J Immunol (2015) 195:488–97. doi: 10.4049/jimmunol.1403225 26056255

[104] Bollyky PL Wu RP Falk BA Lord JD Long SA Preisinger A. ECM components guide IL-10 producing regulatory T-cell (TR1) induction from effector memory T-cell precursors. Proc Natl Acad Sci U.S.A. (2011) 108:7938–43. doi: 10.1073/pnas.1017360108 PMC3093524 21518860

[105] Gagliani N Jofra T Valle A Stabilini A Morsiani C Gregori S. Transplant tolerance to pancreatic islets is initiated in the graft and sustained in the spleen. Am J Transplant (2013) 13:1963–75. doi: 10.1111/ajt.12333 PMC3869180 23834659

[106] Meiron M Zohar Y Anunu R Wildbaum G Karin N. CXCL12 (SDF-1alpha) suppresses ongoing experimental autoimmune encephalomyelitis by selecting antigen-specific regulatory T cells. J Exp Med (2008) 205:2643–55. doi: 10.1084/jem.20080730 PMC2571938 18852294

[107] Pellerin L Jenks JA Chinthrajah S Dominguez T Block W Zhou X. Peanut-specific type 1 regulatory T cells induced in vitro from allergic subjects are functionally impaired. J Allergy Clin Immunol (2018) 141:202–213.e8. doi: 10.1016/j.jaci.2017.05.045 28689791

[108] Gagliani N Amezcua Vesely MC Iseppon A Brockmann L Xu H Palm NW. TH17 cells transdifferentiate into regulatory T cells uring resolution of inflammation. Nature (2015) 523:221–5. doi: 10.1038/nature14452 PMC4498984 25924064

[109] Heinemann C Heink S Petermann F Vasanthakumar A Rothhammer V Doorduijn E. IL-27 and IL-12 oppose pro-inflammatory IL-23 in CD4+ T cells by inducing Blimp1. Nat Commun (2014) 5:3770. doi: 10.1038/ncomms4770 24796719

[110] Roncarolo MG Gregori S Bacchetta R Battaglia M. Tr1 cells and the counter-regulation of immunity: Natural mechanisms and therapeutic applications. Curr Topics Microbiol Immunol (2014) 380:39–68. doi: 10.1007/978-3-662-43492-5_3 25004813

[111] Linterman MA Pierson W Lee SK Kallies A Kawamoto S Rayner TF. Foxp3+ follicular regulatory T cells control the germinal center response. Nat Med (2011) 17:975–82. doi: 10.1038/nm.2425 PMC3182542 21785433

[112] Lee V Rodriguez DM Ganci NK Zeng S Ai J Chao JL. The endogenous repertoire harbors self-reactive CD4+ T cell clones that adopt a follicular helper T cell-like phenotype at steady state. Nat Immunol (2023) 24:487–500. doi: 10.1038/s41590-023-01425-0 36759711 PMC9992328

